# Bibliography

**Published:** 1890-12

**Authors:** 


### BIBLIOGRAPHY.

A TREATISE ON THE IRREGULARITIES OF THE TEETH AND THEIR CORRECTION, including, with the Author's Practice, other Current Methods. Illustrated with nearly 2000 Engravings (not embracing those in the Third Volume). By JOHN NUTTING FARRAR, M.D., D.D.S. Volume I., 758 Pages. New York City, 1888.

The first volume of Dr. Farrar's great work is now ready for distribution, and will soon be followed by two additional volumes. The author has written so voluminously, and has so long been regarded as an authority on regulating teeth, that it may seem perhaps, to many, that they have already thoroughly mastered his ideas, if perchance they may not be able to follow him in all the described practical details. A judgment based on any preceding efforts of the author, the description of which may be found in the periodicals devoted to dentistry, will fail if applied to the present volume. The natural feeling will be that it is a mere collection of these stray papers, with possibly additional comments. Such an opinion would be very far from the truth ; indeed, a perusal of the book indicates that the author has avoided this temptation, and has given the profession a work singularly free in this respect.

In the preface he says, "I have aimed to make my views especially clear, not only to correct current erroneous impressions, but also to place the results of my experience before the profession to whose welfare and progress I have striven to contribute during the best years of my life.

"When I began to practise, principles, methods, and even suggestions in regard to regulating teeth were meagre. The apparatus in use was crude, clumsy, uncleanly, and painful. . . . Invention, to me, has always been a diversion, not a labor, therefore I found enjoyment in this occupation; and when I succeeded in making, among various instruments, devices that seemed to be an improvement on the established forms of regulating apparatus, I thought it no more than right to describe them for the benefit of others."

In the "Preliminary Chapter" the author thus outlines one of the "cardinal principles" of the work: "The importance of the observance of the physiological law which governs tissues during a movement of the teeth (by means of art), the object being to prevent pain. To insure this result (exemption from pain) the pressure by which the movement is to be effected should be under the control of the patient, a requirement which implies the use of instruments capable of being operated and adjusted at will. By this the maximum rate at which it is possible to move teeth painlessly can be ascertained. . . . To determine this rate and test its value, I made a series of experiments which extended over a period of several years. The results were made known to several professional gentlemen in 1873, and in February, 1874, this topic was the subject of my graduating thesis at Jefferson Medical College, and afterwards read, in the main, before the Brooklyn Dental Society, April 12, 1875, and published in the *Dental Cosmos*, January, 1876."

Under the heading, "Scope of the Work," the author says, "This work would have been finished much earlier had there been some accepted classification of subjects to serve as guide. . . . Prior to 1874 very little had been written upon regulation, and that was of a miscellaneous character. There had not been, so far as I know, any attempt to definitely classify mechanical devices; no nomenclature indicating the position of teeth; no classification of expression for individual teeth, nor of types of the arch; little definite concerning harmony between the facial expression and the arrangement of the teeth; no clearly-marked differentiation of the principles adopted in manufacturing appliances, nor any differences recognized in the character of force; no mathematical demonstrations of the philosophy of action and reaction in the bearings of the apparatus on the teeth; no accurate demonstration of the effect of irregularly-developed teeth upon undeveloped ones, nor upon their sockets, by the process of regulating; no definite explanation as to the action of the alveolar tissues under the influence of the different kinds and degrees of force; nor any clear statement as to the difference between moving teeth by causing absorption and moving them by taking advantage of the flexibility of the alveolar tissue. Above all, the fact that pain may be avoided with certainty by conducting the operations within the domain of the physiological functions of tissues was wholly unknown. . . .

"The work is divided, for the convenience of the reader, into several parts. The first volume deals with the history and etiology of the subject: 'The Basal Principles of Regulation;' 'Nomenclature;' 'Principle of Construction of Apparatus;' 'Retaining Devices;' 'Laboratory Rules for Manufacturing Devices;' 'Application of Force;' 'Eruption;' 'Antagonism;' 'Interdental

Spaces;' 'Correction of Irregularities by Grinding, and by Extraction.'

"The second volume contains the 'Classification of Irregularities, and the Various Methods of Treatment for Correction;' 'Straightening Teeth to Line;' 'Turning and Elevating Teeth;' 'Widening and Enlarging the Dental Arch;' 'Correction of Protruding Teeth;' 'Miscellaneous Suggestions, Practical and Theoretical;' and lastly, 'Æsthetics of Dentistry.' . . . The third volume is wholly pictorial, being an object index of all mechanisms described in the other volumes."

It will be seen from these quotations that Dr. Farrar has wisely divided his subject, and takes for preliminary consideration the principles underlying regulation of teeth, and deals largely with the application of force and the construction of the mechanical devices which will best subserve this purpose with a minimum amount of suffering to the patient.

Part II., "History," will repay careful perusal. While many of the facts detailed are familiar to intelligent readers, the author has embodied them into readable form and made them available for reference. He quotes in full from the labors of Dr. Van Marter on the work of the Etruscans, a race of men which preceded the Romans in Italy, and "have been extinct for many years, probably about two thousand;" also interesting extracts from Dr. H. Eames on "Russian Antiquities." He also gives illustrations and descriptions of instruments from Pompeii, which he sketched during a visit to the museum at Naples.

In chapter iii., page 57, he discusses the "History of the Use of the Screw" in regulating teeth, and divides the honor between Dr. W. H. Dwinelle, of the United States, and Charles Gaine, M.R.C.S., of England. To "Dr. Gaine for being the first to use the screw simple, and to Dr. Dwinelle the invention of the screw-jack."

Part III. covers many interesting subjects, among others "The Influences of Evolution upon the Jaws;" "Premature Extraction of Deciduous Teeth;" "Power of Heredity;" "Influences of Insanity and Idiocy upon the Teeth."

In chapter ix., "Upon the Question of Heterogeneousness in the Mixture of Races," the author very ably draws from various sources the conclusion that "there is an underlying power, a law that tends to evolve order out of confusion, not only in mental but also in bodily forms. If study of this law should find a place in the science of dentistry, it would contribute to an education that would lead to broader views." In Part IV. we are introduced to the "Philosophy of the Author's System," and as the views herein expressed underlie the whole of this work they should be given in his own words.

After allusion at length to the "effects of force on teeth" and "tissue-changes by movement of teeth," he remarks: "In regulating teeth by what we will call absorption . . . the tooth is made to plough its own way through the alveolar process, leaving a gap, so to speak, across which the pericemental fibres stretch, which interstitially fills with embryonic matter. This is gradually organized into that which finally becomes secondary alveolar tissue. Perfect reorganization of these tissues requires from one month to a year, sometimes longer, depending upon the age and condition of the patient." Again, on page 159: "Wherever there is tissue with nutrient circulation, there is tissue which serves to convey nerve-force; and where there is nerve-energy there is a substance which is subject to wear and tear. . . . Wherever tracks of nervous energy lead, there are tissues controlled by them; and where there is work, there will be wear and tear of those tissues, and, therefore, need periods of rest. Now, as the act of retrogressive metamorphological change is but a phase of tissue labor, it is easy to see that any such labor caused by moving teeth naturally comes under this head and belongs to this range of phenomena. Hence, in causing absorption, if the degree of force applied be kept within the limit of physiological law, the tissue-changes will be less painful than if the law is violated."

On page 162 the author gives an illustration of this force applied. After trying the most approved elastics for two weeks to move a refractory cuspid, a "very thin metallic band-strap was made to extend round the cuspid and first molar, the ends of the strap being connected on the buccal surface by means of a screw passing through nuts. This screw had sixty threads to the inch, and was shaped at the anterior extremity to fit a watch-key. This simple apparatus (which I denominate a clamp-band) having been applied, the screw was turned so as to cause a slight sense of tightness or pressure, but not enough to cause pain. The sense of tightness passed away within an hour. . . . The screw was turned one-half of a revolution morning and evening, thus advancing it one-half of a thread, or $\frac{1}{120}$ of an inch, by each operation, or $\frac{2}{120}$ of an inch per day. . . . This rate of movement was found to cause no pain. . . . The cuspid continued to advance at this rate daily. . . . This slow advance of the tooth . . . acted so admirably that it was thought best to increase the rate of progress with a view to hurrying the operation, if possible. Accordingly, the screw was turned an entire revolution each morning and evening. . . . This caused some pain, which lasted about three hours, gradually passing away.

"It now became evident that this rate was too rapid; but to make the experiment still more satisfactory in a scientific point of view, the screw was advanced another half-thread each morning and evening, amounting to three revolutions daily. The pain now became almost continuous, accompanied by considerable inflammation."

The conclusion is thus summed up: "1. That in regulating teeth the force should not exceed the bounds of physiological functions. 2. That the plan of moving teeth by elastic materials, though practical, if kept under perfect control, is so difficult to manage that it often leads to pain and inflammation, and is sometimes dangerous to the future usefulness of the teeth, while a properly-constructed apparatus, intelligently operated by means of screws, insures beneficial results without pain or nervous exhaustion. 3. That if teeth are moved by absorption through the alveolar process about $\frac{1}{240}$ of an inch every morning, and the same distance in the evening, no pain or nervous exhaustion follows. 4. That while these tissues will allow an advance of a single tooth at this rate ($\frac{1}{240}$ of an inch) twice in twenty-four hours without exceeding physiological functions, if a much greater pressure be made the changes will become pathological."

The endeavor has been made to select parts clearly indicative of the idea upon which regulation by this method is based. While it will meet, undoubtedly, with opposition in its present form, as it has in the past when presented on the pages of periodicals, it is so clearly founded on well-known physiological laws that it is surprising that any other mode should have ever found favor. It is not understood that Dr. Farrar claims originality in the application of this well-known law of force in connection with tissue; but that he deserves credit for applying it and reducing it to a system no one familiar with the facts can, for a moment, deny. It was taught anterior to Dr. Farrar's active work in the profession, but at no time met with more than a passive recognition. Hence teeth have been moved as rapidly as possible, and the boast has not been infrequent that a regulating case has been completed and patient dismissed in a series of weeks which should have taken, perhaps, an equal number of months. If this portion of Dr. Farrar's work will do something to impress the importance, nay, the absolute necessity of attention to physiological laws, it will have ample reason for having been written, for it is just here, in the opinion of the writer, where there has been the greatest neglect in practical treatment.

Part V. is devoted to "Nomenclature." This briefly gives the plan of the author by which, in his judgment, a better indication of "surface diseases of teeth" may be had.

The text cannot be clearly understood without the diagrams accompanying it. It would seem a mistake to attempt to change the nomenclature which custom has fastened on a profession. It has never yet been attempted with success, any more than the forceful introduction of a manufactured language. Nomenclature is a development, and while oftentimes it may be seriously objectionable, can only be changed by the slow processes of evolution to something better. The plan of the author has the merit of simplicity and is worthy of consideration.

Part VI. will no doubt prove the most interesting to the purely practical mind, as it is concerned with the "Explanation of the Principles in Construction of Regulating Apparatus." Here the author is entirely at home in describing the various devices used in the past and present. The illustrations are copious and most fully explain the text. He devotes one hundred and forty-four pages to describing the simple to the most complex device. While many of these forms have been made familiar by the author, the present arrangement, and, if the expression may be permitted, the dissection of parts, makes the text at once clear and instructive to all classes of mind. It would hardly seem possible that the objection, heretofore made, that these instruments tend to intellectual confusion could be maintained.

Part VII. is devoted to the consideration of "Retaining Devices." These will be found thoroughly described and of great variety of form adapted to peculiar conditions. This, next to the movement of teeth, is the most important feature of regulating. Some novelties are introduced,—novel in the sense of being part of regulating procedures,—one of which is the use of fillings as "retaining plugs." It will, doubtless, be said this is an old operation; but, if so, the writer has failed to observe it used as described.

Part VIII. is confined to the consideration of "Laboratory Rules for Making Regulating Devices." This is exhaustive, covering everything needed for practical work, and will be found invaluable to the dentist who has not had the experience in the working of metals so necessary in the construction of regulating apparatus, and even to those familiar with the work the ideas will be very suggestive.

In this part is also explained the "application of apparatus," a very important matter, and the "retention of devices," being equally valuable, the author devoting considerable space to the "rules" governing it.

Passing over Part IX., on the "Philosophy of the Application of Force," which is a very important part, but which requires the diagrams to clearly explain it, we come to Part X. This is devoted to the "Eruption of Teeth," and Part XI. to "Antagonism of Teeth." Considerable space is given to this important matter in connection with irregularities, and in Part XII., on grinding teeth "improperly locked." The "evening the ends of teeth," and "interfering cusps," will at once recall to the practical mind the ample scope here for original work, and it is made use of by the author to the fullest extent, exhibiting thoroughness of detail and mastery of the subject.

Part XIII., "Interdental Spaces." This difficult matter is considered at length, and, as usual, the author has shown his ability in making his ideas clear by his original illustrations.

In chapter lxv. of Part XIV., on "Extraction of Teeth for the Prevention and Correction of Irregularities," the author expresses his views on "The First Adult Molar," and, after reviewing the extreme opinions for and against the retention of this tooth, says, "It appears to me that there is really but little cause for these acrid discussions, and that the problem can be easily solved. If the tooth is comparatively sound, it is valuable for masticating purposes and should not be extracted except, perhaps, in rare cases, to make room for overcrowded cuspids and bicuspids. . . . On the other hand, if the first molar is badly decayed or is pulpless and so degenerated generally that it is impossible to preserve it for any considerable time, its disposal should be determined by these circumstances." He then continues a further discussion of this topic, but space will not permit more extended quotation.

It would give great pleasure to follow the author chapter by chapter in this most suggestive work; but, as it is, the matter has hardly been touched upon, nor, perhaps, has the reviewer been successful in gleaning the most salient points; but those given have aimed to illustrate the thoroughness of the work done. It is rare to find a book too full of valuable practical thought and experience to review easily; but this is one of this character. The temptation is to quote from every page.

The criticism may be made that Dr. Farrar has written too exhaustively and covered more ground than is required, and in this way made it difficult for students of his peculiar theories to master them. To a certain class of mind it will unquestionably prove a problem. To such the recommendation may be made to discard the argument and philosophy and confine their labors to the purely practical. The author at all these points is peculiarly happy in clearing up a subject that has given so much mental disturbance to some when his earlier articles were first given to the profession.

It is hoped that enough has been said to show that the dentist who neglects to have this volume, and those that are to follow, in his library will fail in that intelligent appreciation of what has been accomplished by one man, and which constitutes a marked advance over anything in its special line that has preceded it in the whole history of dentistry.

This review would be incomplete without allusion to the perfect specimen of book-making that this volume presents. From composition to binding it shows most completely the perfection of art in this direction. Perhaps this could not be otherwise with "The De Vinne Press" imprint, but it is a satisfaction to note the progress made in this direction.

---

Dental Surgery for Medical Practitioners and Students of Medicine, by A. W. Barrett, M.B. (London), M.R.C.S., L.D.S.E., Dental Surgeon to the London Hospital, etc. Second Edition, with Illustrations. P. Blakiston, Son & Co., Philadelphia, 1890.

That this little book of 136 pages has met a want seems evident from the fact that it has reached a second edition. It has, however, the objection common to all such works, that it must fail to reach the end desired, in that it omits, perhaps for the sake of brevity, most important matters, even for those for whom the book is specially intended.

On page 16, on the "Eruption of the Wisdom Teeth," no mention is made of the concealed wisdom tooth, deeply embedded in the inferior jaw. The diagnosis of this is difficult, and the results are as serious as the treatment is simple, that the absence of allusion to it is a decided loss to the general surgeon.

More space than necessary is given to "Irregularities," as the average medical practitioner could not make use of the suggestions, and in all probability would not understand them.

Under "Treatment of Caries," page 51, the following sentence occurs: "If this be not evident [exposure of the pulp], or, if the point of exposure be *very minute*, and the cavity of moderate size, a filling may be inserted." (Italics ours.) This is so at variance with accepted rules of practice that it occasions surprise.

On page 54 the following directions for using arsenious acid will doubtless interest, if it does not instruct: "Equal parts of yellow soap and arsenious acid are to be well worked into a bolus, of which a pellet, as large as the head of a good-sized pin, should be carried on an excavator into the washed and dried cavity. . . . The pellet may be held *in situ* by a plug of wool (cotton), which should be removed after twenty-four hours, and replaced with a wool and mastic (varnish) filling. . . . The pain caused by the action of arsenic is generally severe for *three hours*. . . . After six hours the pain has generally quite departed."

The application of "wool" (cotton) without the addition of "mastic" will strike the average operator as a remarkable proceeding.

On page 61 is the following statement: "Extraction or opening into the pulp-cavity afford the only means of relieving acute periodontitis, and a slight consideration of the cause leading up to this condition will serve to convince of the absolute inutility of applying escharotics, or *counter-irritants, to the gum overlying the affected part*."

On page 82 will be found the following: "It is [pyorrhœa alveolaris] characterized by a condition of *chronic inflammation of the gum*, which becomes tumid, separates from the necks of the teeth, and *secretes an offensive, purulent fluid*."

The space is not at command to quote all the statements which require correction or notice. Those given speak for themselves.

Such books, if written at all, should be condensed into the clearest statement of facts. The idea "that anything will do for a child" has passed away, and the opposite is now accepted that the more exact the statement can be made for the unlearned the better. It is to be presumed that the practice described in the text is based on the first and older idea. This explanation is preferred to the other, that this book represents the prevailing ideas of treatment in vogue at the present time in England.

